# Influence of signal processing strategy in auditory abilities

**DOI:** 10.5935/1808-8694.20130113

**Published:** 2015-10-08

**Authors:** Tatiana Mendes de Melo, Maria Cecília Bevilacqua, Orozimbo Alves Costa, Adriane Lima Mortari Moret

**Affiliations:** a PhD in Sciences; Speech and Hearing Therapist - Bionic Ear Center - Hospital Samaritano.; b Full Professor - University of São Paulo; Coordinator of the Bionic Ear Center - Hospital Samaritano - SP.; c PhD; Professor; Coordinator of the Audiological Research Center - Hospital for Rehabilitation of Craniofacial Anomalies - University of São Paulo. Audiological Research Center - Craniofacial Anomalies Rehabilitation Hospital - University of São Paulo - USP - Bauru (SP) - Brazil.

**Keywords:** cochlear implants, hearing loss, outcome assessment (health care), speech perception

## Abstract

The signal processing strategy is a parameter that may influence the auditory performance of cochlear implant and is important to optimize this parameter to provide better speech perception, especially in difficult listening situations.

**Objective:**

To evaluate the individual's auditory performance using two different signal processing strategy.

**Methods:** Prospective study with 11 prelingually deafened children with open-set speech recognition. A within-subjects design was used to compare performance with standard HiRes and HiRes 120 in three different moments. During test sessions, subject's performance was evaluated by warble-tone sound-field thresholds, speech perception evaluation, in quiet and in noise.

**Results:**

In the silence, children S1, S4, S5, S7 showed better performance with the HiRes 120 strategy and children S2, S9, S11 showed better performance with the HiRes strategy. In the noise was also observed that some children performed better using the HiRes 120 strategy and other with HiRes.

**Conclusion:**

Not all children presented the same pattern of response to the different strategies used in this study, which reinforces the need to look at optimizing cochlear implant clinical programming.

## INTRODUCTION

The multichannel cochlear implant (CI) is the most important advance in the treatment of people with severe and/or profound bilateral hearing loss, who do not benefit from using hearing aids (HA).

However, speech perception results with the CI are closely related to factors such as age at surgery, duration of sensory deprivation, duration of device use, hearing loss etiology and optimization of programming parameters.

One of the important parameters established at the time of device programming is the signal processing strategy, which defines how the CI will transform the acoustic information into electrical stimuli to be transmitted to the auditory nerve. The aim is to provide a high-fidelity electrical representation of the acoustic information captured by the speech processor microphone in order to make speech recognition possible for the CI user, especially in difficult listening situations, such as in the presence of competitive noise.

In Brazil, among the brands of CIs approved for use by the National Agency for Sanitary Surveillance (ANVISA), there is the Advanced Bionics. The first CI device with this brand was implanted in March 1991 for research purposes: the Clarion 1.0, which was approved for commercial distribution in the United States by the Food and Drug Administration (FDA) in March of 1996 for adults, and in June 1997, for children - a device called Clarion 1.2. These devices provide three different signal processing strategies: the Continuous Interleaved Sampling (CIS), the Simultaneous Analog Stimulation (SAS) and the Multiple Pulsatile Sampler (MPS).

A big step for the Advanced Bionics devices was the development of the HiRes speech processing, launched in 2002, which provided more temporal information than the signal processing strategies described above. The high rates of stimulation associated with the extraction of temporal fine structure cues provided by this strategy, result in the transmission of the acoustic signal to the CI user in high resolution mode. According to studies carried out by various international CI centers, HiRes strategy for optimizing the speech perception of CI users of Advanced Bionics is really noteworthy^1^, ^2^, ^3^, ^4^, ^5^.

And in 2006, the company launched the HiRes 120 signal processing strategy, based on the technique of virtual spectral channels in order to provide a more detailed acoustic signal spectral representation, along with the other resources provided by the HiRes strategy.

In the HiRes strategy, the incoming sound is filtered in 16 spectral bands and the energy of each band is extracted and passed on with high rates of stimulation, to a single corresponding electrode. In the HiRes 120 signal processing, the input signal is analyzed in a spectral range of 120 bands, so that subsequently, the energy is simultaneously retransmitted to two adjacent electrodes varying the current level ratio. This allows users of this signal strategy to take advantage of residual hearing in relation to the ability of auditory discrimination, getting spectral information with higher resolution, compared to the HiRes strategy. The increased spectral information, together with fine time resolution - already implemented in HiRes strategy, provides better speech perception results in noise, and music appreciation.

Since its launch, the HiRes 120 has been recommended by the Advanced Bionics as a signal processing strategy used in programming the speech processor for children and adults. In general, the different CI manufacturers instruct speech and hearing experts from the CI team about the signal processing strategy recommended for each device model. However, it is extremely important to have a clinical evaluation of results after CI, in the different signal processing strategies, since sometimes the strategy recommended by the company does not benefit all CI users alike.

In this context, the aim of this study was to analyze and compare the individual results vis-à-vis the performance of speech perception in children using CI, using two different signal processing strategies from the Advanced Bionics HiResolution system.

## METHOD

This study was approved by the Ethics in Human Research (Protocol # 68/2011).

### Subjects

The sample was composed of children with pre-lingual hearing loss, unilaterally implanted with the HiRes 90K devices from Advanced Bionics, aged between 5 and 10 years, from both genders, who had word-recognition-in-the-open auditory skills, according to the hearing categories proposed in 1994^6^ and construction of phrases with four or more elements, as presented in the language categories proposed in 1996^7^. We took off the sample those children who had at least one of the following: partial electrode insertion, refrained from using the CI for a period greater than six months, diagnostic hypothesis of auditory neuropathy spectrum, auditory nerve hypoplasia, and multiple deficiencies associated with loss hearing.

Considering such characteristics, the sample totaled 15 participants. However, four were excluded from the study due to problems with scheduling and return to the CI center where the study was carried out.

Thus, our sample consisted of 11 participants, all users of the HiRes 90K internal component and platinum speech processor. The CI was fitted to the left ear in eight participants, and in the right ear in three. In none of the cases studied there was a history of disorders involving the internal component and all had 16 active intracochlear electrodes.

Regarding the onset of hearing loss, all participants were carriers of congenital hearing impairment, they all had hearing aids prior to the CI and the CI surgery was performed before 3 years of age. With regard to etiology, four subjects were identified with genetic hearing loss and the other seven cases had an idiopathic etiology.

All children who participated in this study were frequent users of CI. Regarding the use of hearing aids in the ear contralateral to the CI, six participants were using this device and five did not use the contralateral HA. All were placed in a hearing and speech treatment program in their cities of origin and were enrolled in regular schools.

Such information, as well as demographic data and other information pertaining to the study were collected from the patients' medical records. The demographics of each participant are listed in the Annex A. To facilitate the identification of participants, they were numbered from 1 (S1) to 11 (S11).

Table 1 shows the sample distribution, measured in terms of age at the time of surgery, the duration of auditory sensory deprivation, the age of the participant at the beginning of this study and the duration of device use (beginning of the study).

**Table 1:** Mean, standard deviation, median, minimum and maximum values (years) relative to the demographics of the 11 participants.

	Mean	SD	Minimum	Median	Maximum
Age of CI	1.80	0.80	0	1.75	2.0
Sensory deprivation duration	1.88	0.80	0.91	1.84	2.0
Age at the beginning of the study	6.67	0.98	5.33	6.41	8.08
Duration of use at the beginning of the study	4.85	0.62	4.00	4.83	6.16

### Instruments and Procedures

The participants were submitted to the procedures of this study, which included: 1) investigation of their audiometric thresholds in the free field, 2) evaluation of speech perception by means of the HINT test in silence and in noise. Both, the audiometric thresholds in free field investigation as well as the assessment of speech perception were performed with participants using only the CI, even those who used hearing aids and CI in their day-to-day.

**Figure 1:**
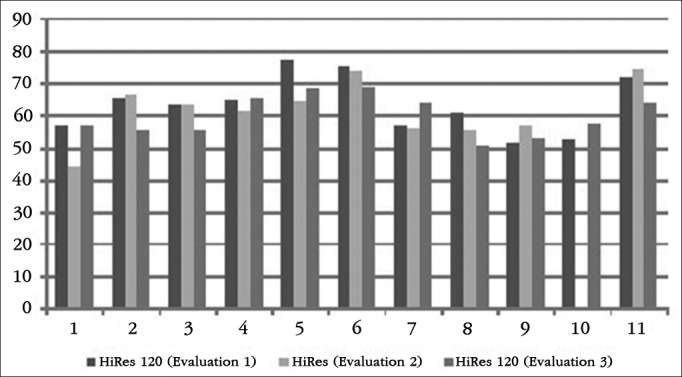
Schematic representation of the evaluation protocol stages.

All procedures listed were fully applied at three different times (Figure 1):

1^st^ Evaluation (baseline): procedures performed using the HiRes 120 strategy. The HiRes 120 strategy was the baseline of the present study, since the strategy was already used by the participants of the study. After this evaluation, we changed the signal processing strategy for HiRes;
2^nd^ Evaluation: After three months of HiRes strategy use, participants were reevaluated by means of the procedures listed above. After the evaluation, we changed the processing strategy for the HiRes 120;
3^rd^ Evaluation: After three months of using the HiRes 120 strategy, the procedures listed were rerun.

Before starting the procedures described below, was assessed the external component (speech processor, transmitter antenna, microphone and wires), through visual inspection, and the internal component was tested using telemetry impedance. The procedures were performed in order to rule out any problem with the device which could interfere in the results presented.

To program the speech processor, at times when we changed the signal processing strategy, we used a portable microcomputer coupled to the programming interface and the programming software from Advanced Bionics - the SoundWave, version 2.0.

After evaluating each sample point, the sound processor was programmed with maps prepared with the signal processing strategy set for each sample point (HiRes or HiRes 120), but the researcher also included in the sound processor the old map of the child, should the child not adapt to the new map. In accordance with the instructions given to the family, the child should use the new maps, adjusted after evaluation, but if the child would not adapted to the new maps, the parents could set the sound processor to the old program (program used before evaluation).

### Audiometric Threshold Study

First, we obtained the audiometric thresholds in free field, for each participant, in the frequencies of 500; 1,000; 2,000 and 4,000 Hz, using the warble tone.

The free field audiometry was carried out in a soundproof booth of 2 x 2 meters and the audiometer used was the Midimate 622 - from Madsen Electronics, connected to an amplifier in free field and two speakers, calibrated in dB - sound pressure level (SPL). The CI user was positioned at one meter from the speaker at 0° azimuth. This procedure was performed to ensure that all participants of the study had proper audibility in the speech frequencies, not compromising the perception tests used in this study.

### HINT test

The HINT test was developed at the House Ear Institute in 1994 to provide a reliable and efficient measurement of speech reception thresholds for sentences in silence and in noise and the Portuguese version of the test - HINT Brazil^8^ was standardized and published in 2008. Since all participants of this study were CI users, the HINT test was introduced only in free field, in silence and with noise coming from the front and lateral to the CI.

In silence, the speech signal was presented via loudspeaker located one meter away and in front of the subject (0° azimuth) at an initial intensity of 65 dB SPL. However, this sign had a variable intensity throughout the procedure, based on the answer given by the patient. This occurred until we established the Sentence Recognition Threshold (SRT), which was achieved when 50% of the sentences were repeated correctly by the participant.

In the frontal noise situation, the speech signal was presented in the presence of noise. Both signals were submitted by the same speaker, located one meter away and in front of the subject (0° azimuth). Under these conditions, the noise was calibrated and maintained at the intensity of 65 dB SPL and the intensity of the speech signal was presented initially at 50 dB SPL. The speech signal varied in intensity throughout the procedure, based on the answer given by the patient. This happened until we established the SRT, which was achieved when 50% of the sentences were repeated correctly by the participant.

In the lateral noise situation, the speech signal was presented in the presence of competitive noise. The speech signal was presented in the front (0° azimuth) and one meter away from the participant, and the noise came from a speaker, positioned at 90° azimuth, ipsilateral to the CI, also one meter away from the subject. Similarly in the frontal noise situation, noise was calibrated and maintained at the intensity of 65 dB SPL and the speech signal intensity was initially presented at 50 dB SPL; however, this signal intensity was variable throughout the procedure, according to the answer given by the subject. This happened until we established the SRT, which was achieved when 50% of the sentences were repeated correctly by the participant.

At the end of the test, the software automatically supplied values in dB in silence (representing the threshold), frontal noise and lateral noise (representing the signal/noise ratio) situations for each participant.

It is Important to stress that the speech stimuli application sequence, in the different situations, occurred randomly in order to eliminate variables related to fatigue, participants' attention and the learning phenomenon. In order to exclude the change variable from the lists in the evaluation of speech perception in different assessment time points in this study, we chose to use the same list of sentences at different time points and in different assessment conditions for each participant.

### Result Analysis Method

The data from all stages of the study were stored in an Excel^®^ database.

To analyze the performance in the three data collection points of our study in the HINT test (silent, noise, front and side noise situations) we used the variance analysis statistical test with repeated measures. For post-hoc comparisons we used the Tukey test.

In both statistical tests used in the present study, we used the STATA computer package, version 9.0 and a significance level of 5%.

## RESULTS

All children evaluated completed the assessment proposed in the three stages of the study, except for child S10. Because the S10 child did not adapt to the signal processing strategy change performed after the first evaluation of the study and continued using the speech processor map set with the HiRes 120 strategy, she was not submitted to the proposed HiRes evaluation strategy. Thus, the results obtained by the S10 child refer only to the auditory performance using the HiRes 120 strategy.

At all the time points evaluated in the study, the children had hearing thresholds with CI less than 30 dB at 500-4000 Hz frequencies. This result ensured that all children had adequate audibility in the speech frequencies, and the audibility of sounds was not a factor which influenced the auditory performance achieved by the participants.

**Graph 1:**
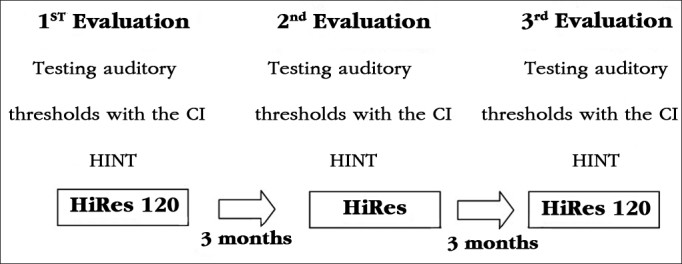
Individual HINT test results - silence. 1: S1; 2: S2; 3: S3; 4: S4; 5: S5; 6: S6; 7: S7; 8: S8; 9: S9; 10: S10; 11: S11.

**Graph 2:**
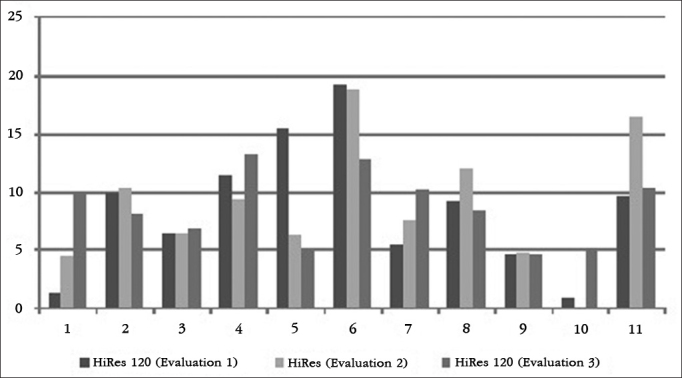
Individual HINT test results - noise in the front.1: S1; 2: S2; 3: S3; 4: S4; 5: S5; 6: S6; 7: S7; 8: S8; 9: S9; 10: S10; 11: S11.

**Graph 3:**
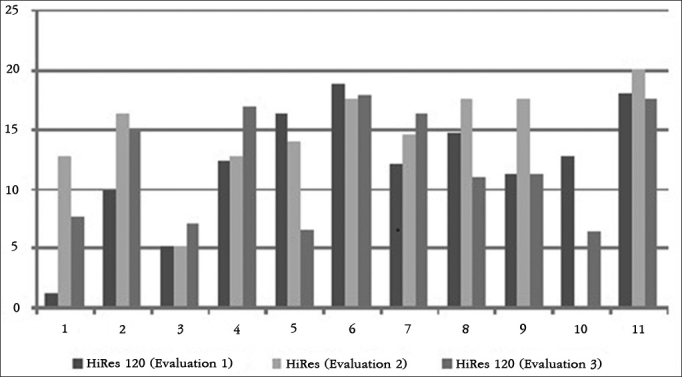
Individual HINT test results - side noise. 1: S1; 2: S2; 3: S3; 4: S4; 5: S5; 6: S6; 7: S7; 8: S8; 9: S9; 10: S10; 11: S11.

The results of each participant in the HINT test, in silence and in frontal and lateral noise can be seen in Graph 1, Graph 2 and 3, respectively.

To facilitate the identification of participants, the column corresponding to the results of each participant was numbered from the participant number 1 to participant number 11.

## DISCUSSION

The signal processing strategy is a programming parameter directly involved in the results of speech perception after CI and, in this context, the present study aimed to analyze and compare the individual results in the speech perception performance in children using CI, using the HiRes and HiRes 120 signal processing strategies, from Advanced Bionics. According to the results obtained, we noticed that not all children had the same response pattern in the different strategies used in this study.

Analyzing auditory performance in silence, some children had better hearing performance with the HiRes 120 strategy (S1, S4, S5, S7), others had better hearing performance with the HiRes strategy (S2, S9, S11). When considering that few active intracochlear electrodes already provide good results in speech recognition in silent environments^9^, it would be expected that the additional spectral information provided by the HiRes 120 strategy would not influence auditory performance in silence.

In the assessment of speech perception in noisy situations, when the noise came from the front, children S2 and S11 had better hearing performance with the HiRes 120 strategy, and children S4, S8 and S9 had better hearing performance with the HiRes strategy. When lateral noise was introduced, children S1, S2, S8, S9 and S11 had better hearing performance with the HiRes 120 strategy and the child S6 had better hearing performance with the HiRes strategy. Considering the results of international studies designed with adult users with post-lingual hearing loss and who showed improvements in hearing performance in noise with the HiRes 120 strategy^10^, ^11^, ^12^, ^13^, due to the increase of spectral information provided by the strategy^9^, it would be expected that the evaluated children had best hearing performance in noise with the HiRes 120 strategy.

The fact that one child (S10) have not adapted to the HiRes is also an important finding of this study, because the child claimed that the sound quality was bad and she could not understand speech when using the program set with the HiRes signal processing strategy.

In order to check whether some patient characteristic influenced the response pattern obtained with different strategies; hearing performance in noise was analyzed in comparison with the characteristics of the participants (Appendix A). However, there were no demographic or hearing characteristic, such as residual hearing (pre-CI hearing thresholds), duration of sensory deprivation, age at CI surgery, age at time of evaluation or duration of CI use that has been instrumental in the response pattern presented by the participant in the study.

It is worth noting in that both situations - silence and the frontal and lateral noisy, some children had a longitudinal improvement in auditory performance (S3, S6, S8 - silence, S5 and S6 - frontal noise; S5 - lateral noise), represented by an SRT reduction during the evaluations, regardless of the signal processing strategy used during the evaluation. This result may be related to the improvement in listening skills. According to the inclusion and exclusion criteria of this study, only children who had hearing ability to recognize open-set words could be selected to participate in it; however, the fact that they had already reached the maximum category, proposed by Geers^6^, does not prevent the CI user to improve hearing skills over time when using the device.

Thus, the results presented in this study reinforce the need to do a clinical assessment when considering post-CI results in the different signal processing strategies, since sometimes the strategy recommended by the company does not benefit all CI users in the say way^11^: some children have better hearing performance using the HiRes strategy, and others have it with the HiRes 120 strategy.

Therefore, it is suggested that CI centers should develop future studies that compare the signal processing strategies of other CI devices in order to investigate the auditory performance of their hearing aid users with the recommended signal processing strategy, and others from a particular device.

## CONCLUSION

According to the results, we noticed that not all children presented the same response pattern vis-à-vis the different strategies used in this study, both in silence and in noise.

## Figures and Tables

**Table:** 

Child	HL etiology	Pre-CI auditory thresholds (without HA)				Pre-CI auditory thresholds (with HA)				Effective use of HA before the CI	HA model
		0.5 Khz	1 Khz	2 Khz	4 Khz	0,5 Khz	1 Khz	2 Khz	4 Khz		
S1	Idiopathic	85	70	85	90	75	70	85	90	Yes	B32 - Widex
S2	Genetic	80	80	80	80	65	60	60	70	Yes	Extra - Phonak
S3	Idiopatic	80	85	↓	↓	65	75	70	80	Yes	Canta 270 - Danavox
S4	Genetic	85	90	90	95	80	80	95	95	Yes	Swift 120 - Oticon
S5	Idiopathic	↓	↓	↓	↓	90	100	↓	↓	Yes	Swift 120 - Oticon
S6	Idiopathic	↓	90	↓	↓	80	90	↓	↓	Yes	Swift 120 - Oticon
S7	Genetic	90	85	95	95	65	65	65	65	Yes	Swift 120 - Oticon
S8	Idiopathic	90	100	100	95	90	90	90	90	Yes	B32 - Widex
S9	Idiopathic	↓	↓	↓	↓	↓	↓	↓	↓	Yes	A675 JSP - Starkey
S10	Idiopática	90	100	↓	↓	75	80	75	↓	Yes	B32 - Widex
S11	Genética	↓	↓	↓	↓	75	85	↓	↓	Yes	Phoenix - Siemens

↓ 100 dB: Missing hearing threshold at 100 dB (equipment at maximum)times/week.											


Child	CI effective used	Using the contralateral HA	Speech and hearing therapy	Weekly frequency of the speech and hearing therapy	Regular school	FM Use
S1	Yes	Yes	Aurioral	2 times/week	Yes	Yes
S2	Yes	No	Aurioral	1 time/week	Yes	Yes
S3	Yes	Yes	Aurioral	2 times/week	Yes	Yes
S4	Yes	Yes	Aurioral	2 times/week	Yes	Yes
S5	Yes	Yes	Aurioral	2 times/week	Yes	Yes
S6	Yes	No	Aurioral	2 times/week	Yes	No
S7	Yes	No	Aurioral	1 time/week	Yes	No
S8	Yes	No	Aurioral	1 time/week	Yes	No
S9	Yes	Yes	Aurioral	2 times/week	Yes	No
S10	Yes	Yes	Aurioral	2 times/week	Yes	No
S11	Yes	No	Aurioral	1 time/week	Yes	No
